# Mechanical properties of tunneling nanotube and its mechanical stability in human embryonic kidney cells

**DOI:** 10.3389/fcell.2022.955676

**Published:** 2022-09-27

**Authors:** Aoqi Li, Xiaoning Han, Linhong Deng, Xiang Wang

**Affiliations:** Institute of Biomedical Engineering and Health Sciences, Changzhou University, Changzhou, Jiangsu, China

**Keywords:** tunneling nanotube, mechanical stress, f-actin, cadherin, cholesterol

## Abstract

Tunneling nanotubes (TNTs) are thin membrane tubular structures that interconnect physically separated cells. Growing evidence indicates that TNTs play unique roles in various diseases by facilitating intercellular transfer of signaling and organelles, suggesting TNTs as a potential target for disease treatment. The efficiency of TNT-dependent communication is largely determined by the number of TNTs between cells. Though TNTs are physically fragile structures, the mechanical properties of TNTs and the determinants of their mechanical stability are still unclear. Here, using atomic force microscope (AFM) and microfluidic techniques, we investigated the mechanical behavior and abundance of TNTs in human embryonic kidney (HEK293) cells upon the application of forces. AFM measurements demonstrate that TNTs are elastic structures with an apparent spring constant of 79.1 ± 16.2 pN/μm. The stiffness and membrane tension of TNTs increase by length. TNTs that elongate slower than 0.5 μm/min display higher mechanical stability, due to the growth rate of F-actin inside TNTs being limited at 0.26 μm/min. Importantly, by disturbing the cytoskeleton, membrane, or adhesion proteins of TNTs, we found that F-actin and cadherin connection dominantly determines the tensile strength and flexural strength of TNTs respectively. It may provide new clues for screening TNT-interfering drugs that alter the stability of TNTs.

## Introduction

Multicellular organisms coordinate cell behavior, regulate morphogenesis, and maintain tissue homeostasis by secreting chemical molecules, releasing exosomes, or establishing intercellular communication such as nerve synapses and gap junctions. In 2004, an intriguing structure termed tunneling nanotube (TNT) that connects long-distance cell pairs was reported (Rustom et al., 2004). They contain a microfilament core, wrapped in a tubular membrane. Numerous studies in the following years have demonstrated that TNTs widely present both *in vitro* and *in vivo* (Cordero Cervantes and Zurzolo, 2021). These thin, straight structures reach lengths from a dozen to tens of microns and hover above the substrate in culture conditions (Austefjord et al., 2014; Zurzolo, 2021). Besides the microfilament-containing thin TNTs, thicker TNTs containing microtubules were also observed in a few cell types (Austefjord et al., 2014). TNTs made up of several individual tunneling nanotubes (iTNTs) were also found recently in neuronal cells (Sartori-Rupp et al., 2019). Although TNTs display heterogeneity in morphology, cytoskeleton composition, and formation (Han and Wang, 2021), they are important subcellular structures that facilitate intercellular communication *via* transferring electrical signals, cellular compounds, and vesicles, which therefore are named the cellular Internet (Baker, 2017).

TNTs not only participate in cellular physiological activities, but also play a unique role in diseases. Our previous study revealed that tumor cells in the early stage of apoptosis are rescued by receiving mitochondria *via* TNTs from neighboring normal cells (Wang and Gerdes, 2015). Similarly, stem cells in lung tissue establish TNTs connection with damaged airway epithelial cells and then transmit mitochondria to repair the epithelial cells (Islam et al., 2012; Ahmad et al., 2014). Also, malignant brain tumor cells establish a TNT network to produce anti-radiotherapy effects (Osswald et al., 2015). Recently, it was found that α-synuclein is transferred between microglia through TNTs to attenuate the inflammatory microglia profile (Scheiblich et al., 2021). Studies have also shown that pathogens use TNTs as a bridge to spread between T cells or nerve cells (Sowinski et al., 2008; Gousset et al., 2009). Therefore, it has been a consensus to explore the treatment of diseases with TNTs as potential targets (Walsh and Selkoe, 2016; Saha et al., 2022).

Reducing the number of TNTs will be a straightforward way to block TNT-dependent communication in TNT-targeted therapy, because the communication efficiency of the TNTs is highly determined by the number of TNTs, i.e., the formation frequency and the lifetime of TNTs (Han and Wang, 2021). The molecular mechanisms of TNT formation have been studied extensively, which are most involved in actin regulators and motor proteins. However, it is difficult to find a broad-spectrum TNT inhibitor because of the heterogeneous mechanisms of TNT formation in different types of cells (Han and Wang, 2021). On the other hand, TNT-dependent communication could be disrupted by mechanical stress (Polak et al., 2015), because TNTs are breakable structures with an average lifetime ranging from a few to tens of minutes in different types of cells (Austefjord et al., 2014; Ariazi et al., 2017). From this point of view, it may be feasible to regulate the number of TNTs by modifying their mechanical stability. Nevertheless, little is known about the mechanical property of TNTs and the key cellular molecules that determine their mechanical stability. Thus in this study, we investigated the mechanical property of TNTs by estimating the elasticity and membrane tension of TNTs. We also investigated key molecules that affect the tensile and flexural strength of TNTs by mimicking the axial and radial load applied to TNTs in tissue. Our results proved that the TNTs are elastic structures whose lifetime is dominated by their mechanical stability.

## Materials and methods

### Microfluidic device fabrication

Silicon-master with a straight channel pattern (depth = 50 μm, width = 400 μm, length = 10 mm) was fabricated by deep reactive ion etching (YW MEMS, China). Polydimethylsiloxane (PDMS) microfluidic chips were molded in a MicCell casting station (GeSiM GmbH, Germany) by injecting pre-mixed (10:1 ratio of base to curing agent) Sylgard 184 elastomer (Dow Corning, United States) between the Si-master and a polycarbonate lid. Then the assembled device was thermally cured at 65°C for 4 h and cleaned with water. After drying, the pattern surface of the PDMS was treated with oxygen-plasma (PT-5S, Sanhe Boda Electromechanical Technology, China), and placed onto a glass coverslip to seal the channel.

### Cells and in-chip culture

Human embryonic kidney cells (HEK293, ATCC CRL-1573) were cultured in Dulbecco’s Modified Eagle Medium (Gibco, Life Tech, United States) supplemented with 10% fetal calf serum (Gibco) at 37°C and 5% CO_2_. To visualize Cavin-1 in live cells, HEK293 cells were transfected with Cavin-1-mEGFP (gift from Ari Helenius, Addgene plasmid # 27,709) by Lipofectamine 3000 transfection reagent (Thermo Fisher Scientific, United States) according to the manufacturer’s recommendations and further selected by adding 400 μg/ml G418 (Sigma-Aldrich, United States) for screening and 200 μg/ml for maintenance. To visualize actin, HEK293 cells were transiently transfected with LifeAct-TagGFP2 plasmid (Ibidi GmbH, Germany).

After chip assembling, the channel was primed with 70% ethanol and washed with sterilized water. Then the channel was coated with 50 μg/ml fibronectin (Sigma-Aldrich) to facilitate cell adherence. Afterward, a small volume (5 μl) of dense cell suspension (2×10^6^ cells/ml) was slowly injected into the inlet of the channel, and left to passively flow through the channel. After 2 h, unattached cells were removed by gently replacing the medium. Then, the chip was left to stabilize in the incubator overnight.

### Experimental setup and flow control

The microfluidic chamber with cells was mounted onto a motorized stage (Prior Scientific Inc., United States) of a Zeiss Axio Observer 7 fluorescence microscope (Carl Zeiss MicroImaging, Germany) equipped with a 40×/0.16NA objective and an ORCA-Flash4.0 camera (Hamamatsu, Japan) with 37°C and 5% CO_2_. The outlet was connected to a piezo-controlled microsyringe pump (Cetoni GmbH, Germany). A 2.5 ml syringe was controlled by the pump to withdraw fluid to produce a defined accurate volumetric flow rate in the microfluidic channel. For drug treatments (all from Sigma-Aldrich), 100 µM ethylene glycol-bis(2-aminoethylether)-N,N,N′,N′-tetraacetic acid (EGTA), 100 µM methyl-beta-cyclodextrin (Mbc), or 50 nM cytochalasin D (cytoD) was used. Medium containing the drug was introduced into the channel by withdrawing the medium from the outlet at a flow rate of 0.01 μl/s to ensure the channel was filled with the drug. After 18-min incubation, a 2-min pulse hydrodynamic treatment at indicated flow rate was performed.

### Imaging and quantification of TNTs

To record the bending of TNTs, a LifeAct-positive TNT located vertically in the direction of the flow was selected. A flow of 0.1 μl/s was held for 10 s to await static equilibrium in the bent TNT, then the flow was withdrawn. Fluorescence images were acquired by the ZEN software (Carl Zeiss MicroImaging). To quantify the number and orientation of TNTs in the channel, more than 20 areas were randomly selected for bright-field live-cell imaging (5 or 30 min/frame) using the multi-position function. The number, length, and angle of TNTs at indicated time points were measured manually using NIH ImageJ. The percentage of residual TNTs was calculated as the number of TNTs remaining after treatment to that before treatment in the same population of cells. The elongation rate of a TNT was calculated according to the changes in the length of TNT between two frames (5 min).

### AFM indentation experiment

HEK293 cells were cultured on a 3-cm Petri dish overnight. Then, the medium was replaced with CO_2_-independent medium (Franz et al., 2007). AFM indentations were performed using a NanoWizard 3 setup (JPK Instruments, Germany) mounted on a Zeiss inverted microscope with a controlled temperature of 37°C. The tip of a cantilever (k = 0.1 N/m, MLCT-O10, Bruker, Germany) was positioned 3–5 µm over a TNT. Then, the center of the TNT was indented by the cantilever with a trigger force of 0.3 nN at a rate of 2 μm/s. The resulting force-distance curves were collected using the JPK data processing software. To calculate the apparent spring constant of TNTs, the ratio of vertical deflection force (pN) to tip-sample indentation distance (µm) was calculated according to the slope of the force-distance curve during TNT indentation automatically generated by the Elasticity Fit function of the JPK software. To ensure the right events were recorded, only distance-force curves showing all these stages including flat (approach the TNT), inclined (indentation the TNT), and steep (reach the substrate) stage were used for analysis. Eventually, eight out of all the tested TNTs fulfilled such criteria.

### Fluorescence staining and measurement

Cultures of cells in μ-Slide 8-well chambers (Ibidi) were fixed with 4% paraformaldehyde (Sigma-Aldrich) in PBS 24 h after plating. Immunofluorescence staining was performed according to standard procedures by using primary antibodies: mouse anti-caveolin-1 (ab17052, Abcam), mouse anti-N-cadherin (610,920, BD Transduction Laboratories), mouse anti-E-cadherin (ab76055, Abcam), rabbit anti-β-catenin (C2206, Sigma-Aldrich), mouse anti-α-tubulin (Sigma-Aldrich), and mouse anti-vimentin (ab8978, Abcam), followed by secondary antibody (Thermo Fisher Scientific): goat anti-mouse AF546 and donkey anti-rabbit AF488. F-actin was stained with tetramethylrhodamine (TRITC) conjugated phalloidin (Yeasen Biotech, China) or phalloidin-AF647 (Thermo Fisher Scientific). Nucleus were labeled by using Hoechst 33,258 (HO, Sigma-Aldrich) or propidium iodide (PI, Sigma-Aldrich). Membrane glycoproteins were labeled by wheat germ agglutinin (WGA) conjugated Alexa Fluor 488 or 633 (Thermo Fisher Scientific). For membrane cholesterol staining, fixed cells were incubated with filipin (1:500 in PBS, Sigma-Aldrich) for 2 h at room temperature. All the fluorescence-labeled samples were imaged using a Zeiss 710 confocal microscope equipped with a 20×/0.5NA or 40×/0.75NA objective (Carl Zeiss MicroImaging).

The abundance of Cavin-1 or WGA-AF488 on TNTs was assessed by quantifying the fluorescence intensity. Cavin-1-EGFP positive or WGA-AF488 stained live cells were imaged at 5 min per frame over a period of 25 min using the confocal microscope equipped with the ×40 objective. Obtained images were analyzed by using ImageJ. Briefly, a 1-μm length located in the middle of a TNT was selected as the region of interest (ROI). The cytoplasmic region of TNT-connected cells was selected to determine the basement fluorescence. The relative fluorescence intensity (RFI) is defined as RFI = F_t_/F_c_, where F_t_ is the mean fluorescence intensity of the ROI, F_c_ is the basement fluorescence intensity acquired from the TNT-connected cell in the same image.

### Analysis of F-actin flow

Analysis of F-actin flow was performed on the Zeiss 710 confocal microscope with a 63×/1.40NA objective and the fluorescence recovery after photobleaching (FRAP) module. An ROI was chosen as a circle (4 μm) in the middle of a LifeAct-positive TNT. Before bleaching, the TNT region was imaged for 30 s to obtain a baseline. Local photobleaching was achieved by the repetitive illumination of the ROI with argon laser (488 nm) at full power. After bleaching, the TNT region was imaged for 180 s. Recorded time-lapse sequences were exported uncompressed and kymographs were analyzed with ImageJ. The rate of F-actin flow was calculated according to the movement distance of the bleached region within 180 s.

### Statistical analysis

Each experiment was performed with at least three groups of independent samples. Data were analyzed with Pearson’s chi-squared test using SPSS (IBM, United States) or Student’s two-tailed *t*-test (Microsoft Excel, United States). Values are expressed as mean ± S.D. All error bars show 95% confidence intervals. *, ** and *** indicate *p* < 0.05, 0.01 and 0.001, respectively.

## Results

### The elastic property of TNTs

**FIGURE 1:**
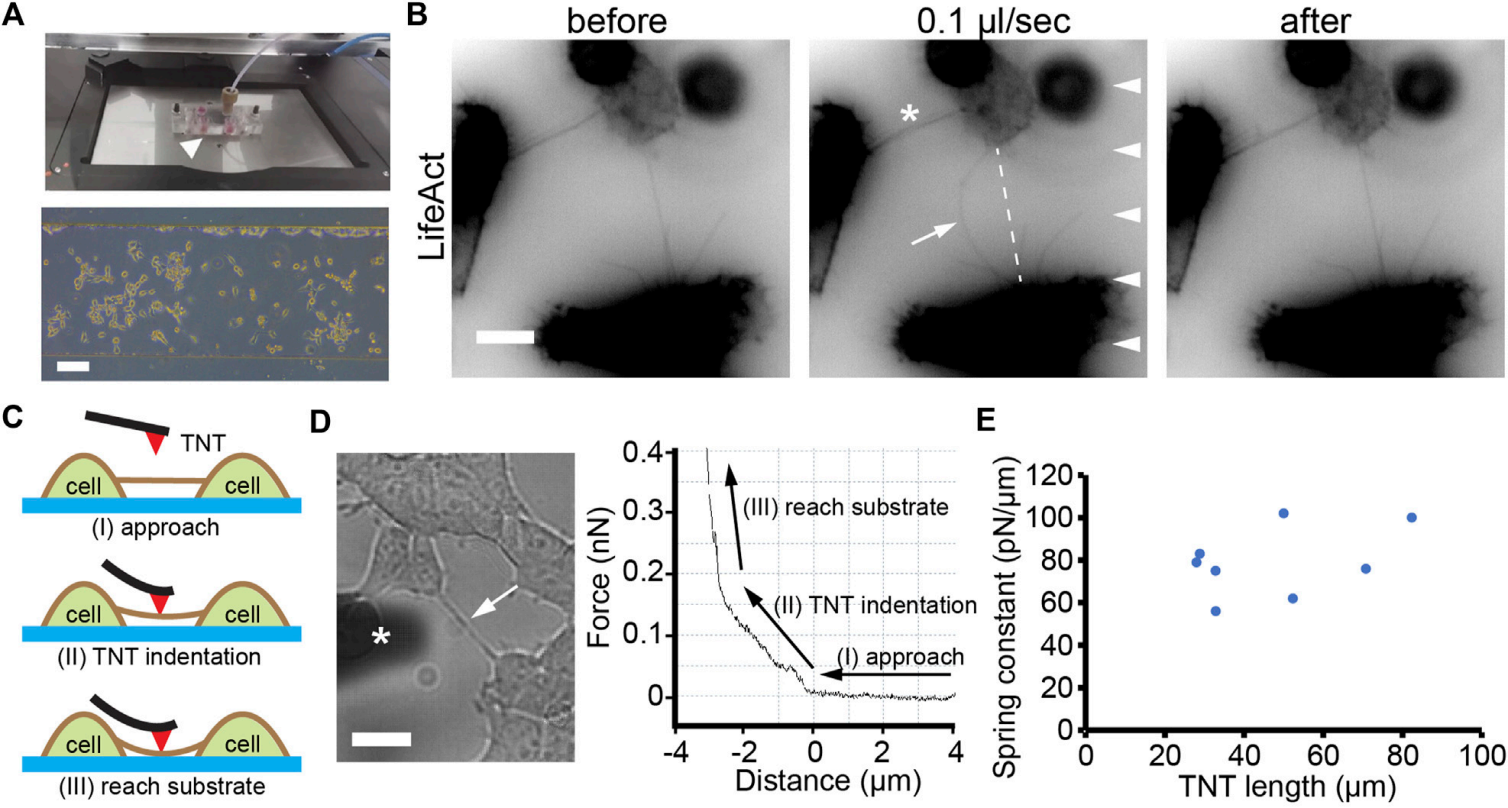
Elastic property of TNTs. **(A)** Top: Microfluidic setup (arrowhead) mounted onto the stage of microscopy. Bottom: phase contrast image of cells after 12 h of culturing inside a microfluidic channel. Scale bar, 100 μm. **(B)** A TNT (arrow) between two LifeAct-positive HEK293 cells bend when exposed to laminar flow (0.1 μl/s, arrowheads) and recovered when the flow stopped. Note that the other TNT (asterisk) did not bend due to the small angle between the TNT and the direction of flow. Scale bar, 10 μm. **(C)** Schematic illustration of the AFM indentation experiment showing three stages: approach, TNT indentation, and reach substrate. **(D)** A TNT (arrow, left figure) under an AFM cantilever (marked with an asterisk). The force-distance curve includes the three stages. Scale bar, 10 μm. **(E)** Apparent spring constants of TNTs with different lengths.

To explore the mechanical property of TNTs, we monitored the deformation of TNTs by applying radial force on TNTs using laminar flow generated in a microchannel (Figure 1A). To ensure that the TNTs are subjected to the hydrodynamic force, HEK293 cells were used because these cells are morphologically round with a height of about 20 μm, so they form TNTs hovering at a certain distance above the substrate, as opposed to those thin flat cells such as fibroblasts and epithelial cells. HEK293 cells expressing LifeAct were cultured in the channel overnight to form TNTs. As shown in Figure 1B, a TNT displayed straight in the static condition. When the medium was pumped into the channel at a low flow rate (0.1 μl/s, 4.3 dyn/cm^2^), the TNT bend and reached a steady state in the direction of the laminar flow. After the cessation of flow, the TNT resumed its original form, indicating that it was an elastic structure. It should be noted that LifeAct fluorescence in the TNT was continued during bending without gaps or depletion, suggesting actin filaments entered into the elongated TNT.

To further quantitatively analyze the elasticity of TNTs, we used an AFM to measure the spring constant of TNTs (Figure 1C). The advantage of the high location of TNTs in HEK293 cells ensures a meaningful distance of vertical deformation during indentation. As shown in Figure 1D, When the AFM cantilever indented to the middle position of a TNT, the force continually increased with distance, suggesting the TNT bent but did not break during the indentation. Then, the force-distance curve suddenly became steep due to the cantilever reaching the rigid glass substrate. Since TNTs are thin tubular structures that hover above the substrate, they are neither homogeneous nor purely elastic. Thus, we here adopted spring constant (i.e., the ratio of indentation force and vertical deflection) but not elasticity modulus to character the elastic property of TNTs. The calculation reveals that the apparent spring constant of TNTs in HEK293 cells is 79.1 ± 16.2 pN/μm (*n* = 8, Figure 1E).

### The membrane tension of TNTs

**FIGURE 2:**
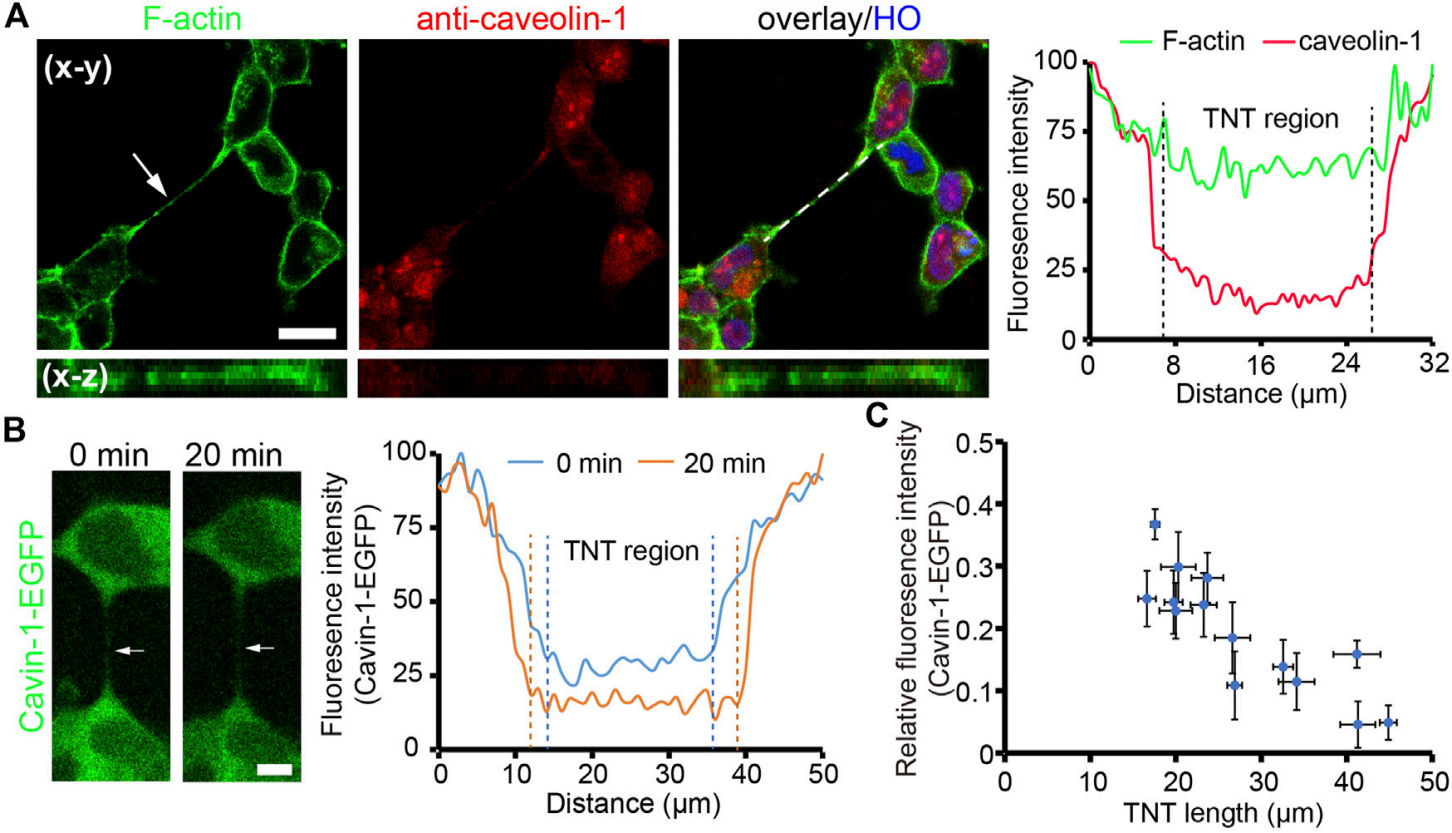
Tension of TNT membrane. **(A)** Localization of caveolin-1 on a TNT (arrow). Caveolin-1 proteins were immunostained with anti-caveolin-1 antibody and imaged by the confocal microscope. The right panel shows the fluorescence intensity profile of caveolin-1 and F-actin staining along the dashed line. **(B)** Distribution of cavin-1-EGFP during TNT elongation. The intensity profile demonstrates a reduced abundance of cavin-1-EGFP on a TNT (arrow) when the TNT elongated after 20 min. **(C)** Relative fluorescence intensity of cavin-1-EGFP on the center of TNTs with different lengths during 25 min live-cell imaging. Scale bars, 10 μm.

Either straight or bent TNTs observed indicate that TNTs need to maintain a certain level of tension to resist the stretch generated by cells. Here, we focused on the membrane tension of TNTs by measuring the abundance of caveolar marker proteins on TNTs. When the membrane tension increases, the caveolar structure on the membrane surface disassembles, resulting in reduced distributions of Cavin-1 and caveolin-1 as a punctuated pattern, two caveolar marker proteins (Sinha et al., 2011). We first examined the existence of caveolin-1 on TNTs by using immunofluorescence. As shown in Figure 2A, there was very little abundance of caveolin-1 on the TNT region, probably due to reduced/absence of caveolar invaginations on the TNTs, as TNT membranes intended to be stretched. To further verify the high membrane tension of TNTs, we used HEK293 expressing Cavin-1-EGFP and analyzed the distribution of the protein on TNTs. Consistent with the observation of immunofluorescence of caveolin-1, Cavin-1-EGFP was less on the TNT membrane than on the plasma membrane (Figure 2B; Supplementary Movie S1). More importantly, there was a decreasing fluorescence intensity of Cavin-1-EGFP when the length of TNT increased by cell dislodgement. Further statistical analysis revealed the fluorescence intensity of Cavin-1-EGFP was lower in longer TNTs (*n* = 14, Figure 2C; Supplementary Figure S1A), while the abundance of membrane tension insensitive glycoproteins on TNTs did not display such tendency (*n* = 21, Supplementary Figure S1B). The results suggest that the membrane tension of long TNTs is higher than short TNTs.

### The mechanical stability of TNTs

**FIGURE 3:**
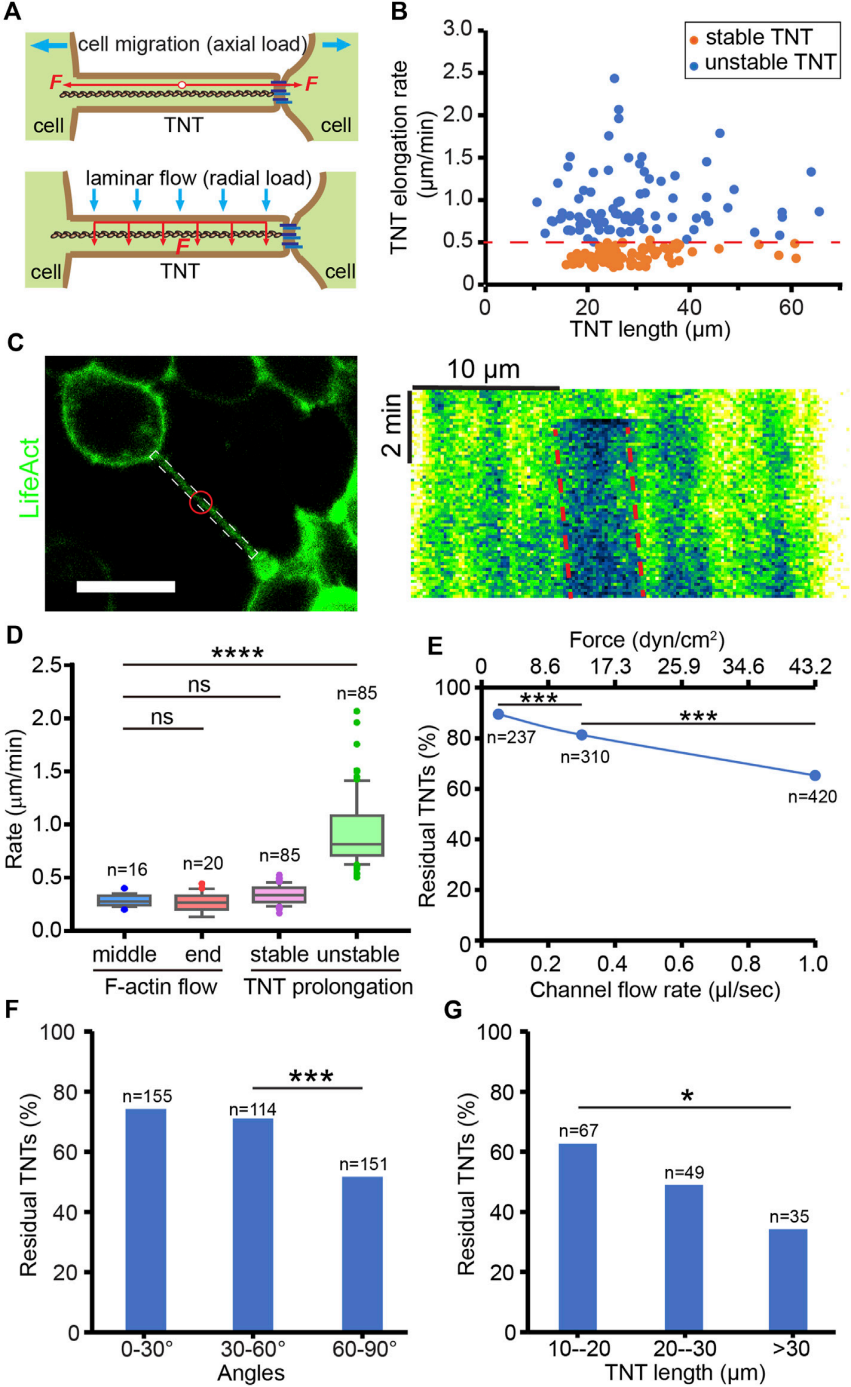
Mechanical stability of TNTs. **(A)** Schematic diagram of the axial and radial load of a TNT. **(B)** Elongation rate of stable and unstable TNTs induced by TNT-connected cell migration. **(C)** Analysis of F-actin flow in a LifeAct-positive TNT. Red circle indicates the bleached area. Scale bar, 20 μm. Kymograph generated from the white boxed area in the left image shows the movement of the bleached area (red dash lines) in the TNT, where the x-axis is the scale bar of length, and the y-axis is the scale bar of time. **(D)** Comparison of the flow rate of F-actin in the middle and end position of TNTs and the elongation rate of stable and unstable TNTs. **(E)** The percentage of residual TNTs after different flow stress treatments. **(F)** Effect of flow stress (1 μl/s) on the number of TNTs with different angles. **(G)** Longer TNTs (60–90°) are more mechanically sensitive to flow stress (1 μl/s).

The number of TNTs is not only determined by the frequency of formation, but also by their mechanical stability since TNTs prone to break under overload tension. Here, the mechanical stability of TNTs was studied by evaluating their tensile strength and flexural strength, which respectively resist the axial load derived from TNT-connected cell pairs undergoing migration and radial stress from the microenvironment (Figure 3A). The formation and maintenance of TNTs in HEK293 cells always experienced axial force deriving from cell dislodgement (Supplementary Movie S2). To investigate the tensile strength of TNTs, we analyzed the elongation rate of TNTs that is dependent on the axial load from cells by measuring the length changes of TNTs at different time points. TNTs keeping stable for at least 5 min after measurement herein are referred to as “stable TNTs”, and TNTs that break within 5 min after measurement are “unstable TNTs” (Supplementary Figure S2). The elongation rate of stable TNTs was significantly lower than that of unstable TNTs (0.35 ± 0.08 vs. 0.95 ± 0.37 μm/min, *p* < 0.0001, *n* = 85) with a threshold rate of approx. 0.5 μm/min (Figure 3B). Moreover, we found that the elongation rate of both stable and unstable TNTs did not show significant relevance to the length of TNTs. This suggests that the ability of TNTs to bear axial force is length-independent. Considering the prolongation of TNTs requires the growth of F-actin inside, we measured the movement of F-actin in TNTs by bleaching LifeAct (Figure 3C). The movement of bleached actin was 0.26 ± 0.07 (*n* = 16) and 0.26 ± 0.09 μm/min (*n* = 20) at the middle and end position of TNTs respectively, which displays that the F-actin flow rate was no different from the elongation rate of stable TNTs, but significantly slower than the elongation rate of unstable TNTs (Figure 3D). It suggests that F-actin growth may be a critical factor in determining the stability of TNTs under axial loading.

To mimic the mechanical stress applied in the radial direction of TNTs, we applied hydrodynamic force on TNTs by using the microfluidic channel. Cells were treated by laminar flow for only 2 min to minimize the effect of axial load due to cell migration. By counting the number of TNTs before and after different loads of flow-induced stress (2.16 dyn/cm^2^ at 0.05 μL/s, 12.96 dyn/cm^2^ at 0.3 μL/s, 43.2 dyn/cm^2^ at 1.0 μL/s), we found that the percentage of residual TNTs decreased as the stress increased (chi-square, *p* < 0.0001, Figure 3E). Since the TNTs observed in the channel were randomly oriented, we further divided them into three groups. As shown in Figure 3F, only TNTs oriented 60–90° to the flow had a significantly reduced residual rate (chi-square, *p* < 0.0001), due to being subjected to greater hydrodynamic force. Therefore, we selected TNTs that orient to the flow at 60–90° in the following experiments. In this group, TNTs shorter than 20 µm in length were more stable than those longer than 30 µm when treated with 1.0 μL/s flow (chi-square, *p* < 0.05, Figure 3G), indicating that the flexural strength of short TNTs is higher than that of long TNTs.

### The molecular determinant of TNTs’ mechanical stability

**FIGURE 4:**
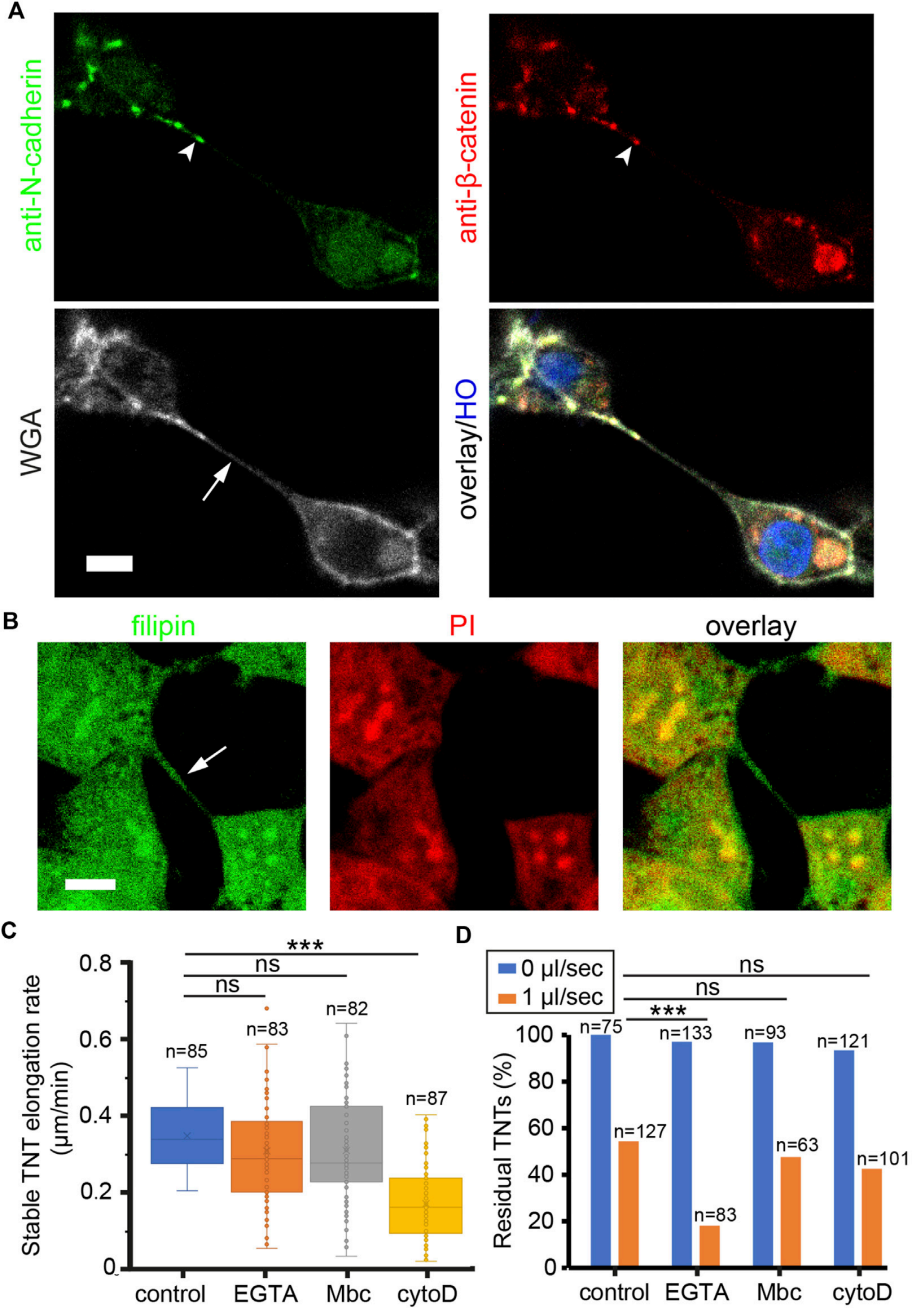
Molecular components of TNTs regulate their mechanical stability. **(A)** Co-localization of N-cadherin and β-catenin (arrowheads) at the tip of TNT. TNT (arrow) was stained with WGA-AF633. Nucleus were labeled by HO. **(B)** Cholesterol on TNT (arrow) and plasma membrane was stained by filipin. Nucleus were labeled by PI. **(C)** Effect of drugs on the elongation rate of stable TNTs. **(D)** Effect of drugs on the number of TNTs in static and flow stress (1 μl/s) conditions. Scale bars, 10 μm.

To identify which molecular component of TNTs dominantly determines their mechanical stability, we focused on the molecules associated with cytoskeleton, adhesion, and membrane lipid of TNTs. The cytoskeleton of TNTs in HEK293 cells is composed of F-actin without microtubules and vimentin (Supplementary Figure S3A). It has been shown that cadherin adhesion molecules are present at the tip of TNTs or between thin TNTs (Jansens et al., 2017; Sartori-Rupp et al., 2019; Chang et al., 2022). Here, we show that HEK293 cells expressed N-cadherin but not E-cadherin at cellular junctions (Supplementary Figure S3B). Immunofluorescence imaging demonstrates co-localization of N-cadherin and β-catenin towards the end of WGA labeled TNT (Figure 4A), suggesting that N-cadherin may be the anchor molecule docking TNT to the neighboring cell. For the membrane study, cholesterol was stained by filipin fluorescent probe since it is the key molecule affecting membrane stiffness and tension (Chakraborty et al., 2020). Confocal imaging shows that both the cell and TNT membrane contained abundant cholesterol (Figure 4B).

To destabilize TNTs by interfering with these molecules, a dose titration test was first performed to determine the minimal concentration of drugs in reducing the number of TNTs (Supplementary Figure S4). Next, cells were pre-treated with 50 nM cytoD to inhibit actin polymerization, 100 µM EGTA to abrogate cadherin interactions, or 100 µM Mbc to extract cholesterol from the membrane. After 20 min, the elongation rate of stable TNTs was measured as shown in Figure 4C. The elongation rate that TNTs could tolerate was significantly reduced in cells treated with cytoD. Together with the result that the F-actin flow rate in TNTs decreased in the presence of 50 nM cytoD (Supplementary Figure S5), it indicates that the growth rate of F-actin is essential for the tensile strength of TNTs. In the study of flexural strength, cells in the channel were pre-treated with the drugs, and kept in static condition or treated with a flow of 1 μL/min for 2 min. The number of TNTs before and after the flow stress was counted. The result reveals that under hydrodynamic pressure, EGTA treatment had a significantly higher inhibitory effect on the residual rate of TNTs as compared to other treatments (chi-square, *p* < 0.001, Figure 4D), suggesting that the N-cadherin interactions are critical to the flexural strength of TNTs.

## Discussion

The communication efficiency of TNTs is largely dependent on the number of TNTs which is determined by the frequency of TNT formation and the lifetime of TNTs. As mechanical sensitive structures, the limited lifetime of TNTs is greatly influenced by mechanical stress. However, the mechanical property and stability of TNTs have not been well studied. Here, our work confirmed that TNTs are elastic structures that bend under radial force. Indeed, *in vivo* observations show that curved TNT-like structures present in brain and embryo tissue (Osswald et al., 2015; Alarcon-Martinez et al., 2020; Scheiblich et al., 2021), indicating that they tolerate radial forces derived from adjacent cells and extracellular matrix. Furthermore, we measured the spring constant of TNTs (79.1 ± 16.2 pN/μm). It is close to the lateral force (approx. 40 pN/μm) that is needed to bend TNTs between Hela cells using optical tweezers (Chang et al., 2022). Moreover, both studies revealed no threshold was required for bending TNTs. We also observed the continuity of F-actin inside bending TNTs, suggesting that actin filaments are assembled to accommodate the elongation of TNTs.

Molecular dynamics simulations on membrane nanotube pulling behaviors reveal that further increase of either the force or the pulling velocity causes the nanotube rupture (Li et al., 2019). Our result demonstrated that the stability of TNTs is very sensitive to the pulling velocity, i.e. the separation speed of cell pairs. Those cells that separate faster than 0.5 μm/min are prone to break TNT connections. Thus, the lifetime of TNTs in different types of cells varies greatly probably due to the differences in cell migration velocity. Importantly, by comparing the rate of F-actin flow with the elongation rate of TNTs, we found that the movement of F-actin is similar to the elongation rate of stable TNTs, but much slower than that of unstable TNTs. It is concluded that the growth rate of F-actin is the rate-limiting factor in TNT elongation.

The study on the distribution of membrane tension-sensitive proteins on TNTs indicates that the tension in long TNTs is higher than that in short TNTs. Therefore, longer TNTs are more rigid and bear higher tension from TNT-connected cells. Moreover, by evaluating the mechanical stability of TNTs under either axial or radial stress, we found that the flexural strength of long TNTs is less than that of short TNTs particularly when the flow shear stress is higher than the range of physiologic shear stress in the microvasculature (<10 dyn/cm^2^) (Polacheck et al., 2019), probably due to longer TNTs bearing higher radial stress. Thus, the mechanical instability of long TNTs impedes the distance of TNT-dependent communication.

In the study exploring the molecular determinant of mechanical stability of TNTs, we found that the elongation rate of TNTs reduced greatly when actin filament polymerization was inhibited by cytoD, suggesting again that actin cytoskeleton is the main component providing tensile strength of TNTs. Cadherin has been found in both open-ended and close-ended TNTs (Sartori-Rupp et al., 2019; Chang et al., 2022). Cadherin proteins may provide a binding force to anchor the two membranes for maintaining the connections at the tips of TNTs (Jansens et al., 2017; Sartori-Rupp et al., 2019). Here, our calcium depletion experiment revealed that EGTA treatment led to a decrease in the mechanical stability of TNTs, which could not tolerate high flow stress. So the resistance of the cadherin interactions to radial stress is the key factor that determines the flexural strength of TNTs. Nevertheless, KO/KD experiments including rescue experiments are further needed to underlie the role of cadherin in TNT-cell adhesion. Considering cadherin may be a potential target in developing efficient and safe drugs abolishing TNT connection, future work is focused on testing the effect of cadherin antagonists, such as synthetic linear peptides, cyclic peptides, and non-peptidyl peptidomimetics (Yu et al., 2019), on the number of TNTs. In addition, a wider investigation should be carried out to study the mechanical properties of microtubule-positive TNTs and TNTs composed of a bundle of iTNT. In conclusion, we established methods to characterize the mechanical properties of TNTs and evaluate their mechanical stability. The study on the tensile and flexural strength of TNTs helps us understand the lifetime and effective distance of TNTs in tissue’s complex mechanical microenvironments. Moreover, the exploration of the role of the TNT component in the stability of TNTs may provide new clues for the development of drugs targeting TNTs.

## Data Availability

The original contributions presented in the study are included in the article/Supplementary Material, further inquiries can be directed to the corresponding authors.

## Supplementary material

SUPPLEMENTARY FIGURE S1 Distribution of cavin-1-EGFP and glycoprotein on short and long TNTs. **(A)** Abundance of cavin-1-EGFP on a short and a long TNT (arrows). The relative fluorescence intensity is the ratio of the fluorescence intensity of the TNT to that of the TNT-connected cell. **(B)** Representative images of staining of short and long TNTs with WGA-AF488. Right: relative fluorescence intensity of WGA-AF488 on the center of TNTs with different lengths. Scale bars, 10 μm.

SUPPLEMENTARY FIGURE S2 TNT breaks during cell migration.A TNT (arrow) broke (00:10) when the distance between the TNT-connected cell pair increased due to cell migration. Scale bar, 20 μm.

SUPPLEMENTARY FIGURE S3 Cytoskeletal composition of TNTs and cadherin expression in HEK293 cells. **(A)** TNTs (arrows) did not contain microtubules (top) and vimentin (bottom). **(B)** HEK293 cells expressed N-cadherin but not E-cadherin at cellular junctions (arrowheads). Scale bars, 20 μm.

SUPPLEMENTARY FIGURE S4 Effect of different concentrations of drugs on the number of TNTs.Cells cultured in Ibidi chambers were treated with EGTA (10, 50, 100, and 500 μM), Mbc (1, 10, 100, and 500 μM), and cytoD (10, 20, 50, and 100 nM) separately for 60 min. The number of TNTs was counted before and after the treatment.

SUPPLEMENTARY FIGURE S5 Treatment of cytoD inhibits the F-actin flow rate in TNTs.Cells were treated with 20 or 50 nM cytoD for 30 min. The flow rate of F-actin in the middle position of TNTs was measured.

SUPPLEMENTARY MOVIE S1 Life-imaging of cavin-1-EGFP on a TNT in Figure 2B.

SUPPLEMENTARY MOVIE S2 Elongation of a TNT during cell migration.
