# Supplementary figures and images for: Mechanical properties of tunneling nanotube and its mechanical stability in human embryonic kidney cells
Source: Front Cell Dev Biol. 2022 Sep 27;10:955676. doi: 10.3389/fcell.2022.955676 (PMC9551289; doi:10.3389/fcell.2022.955676)

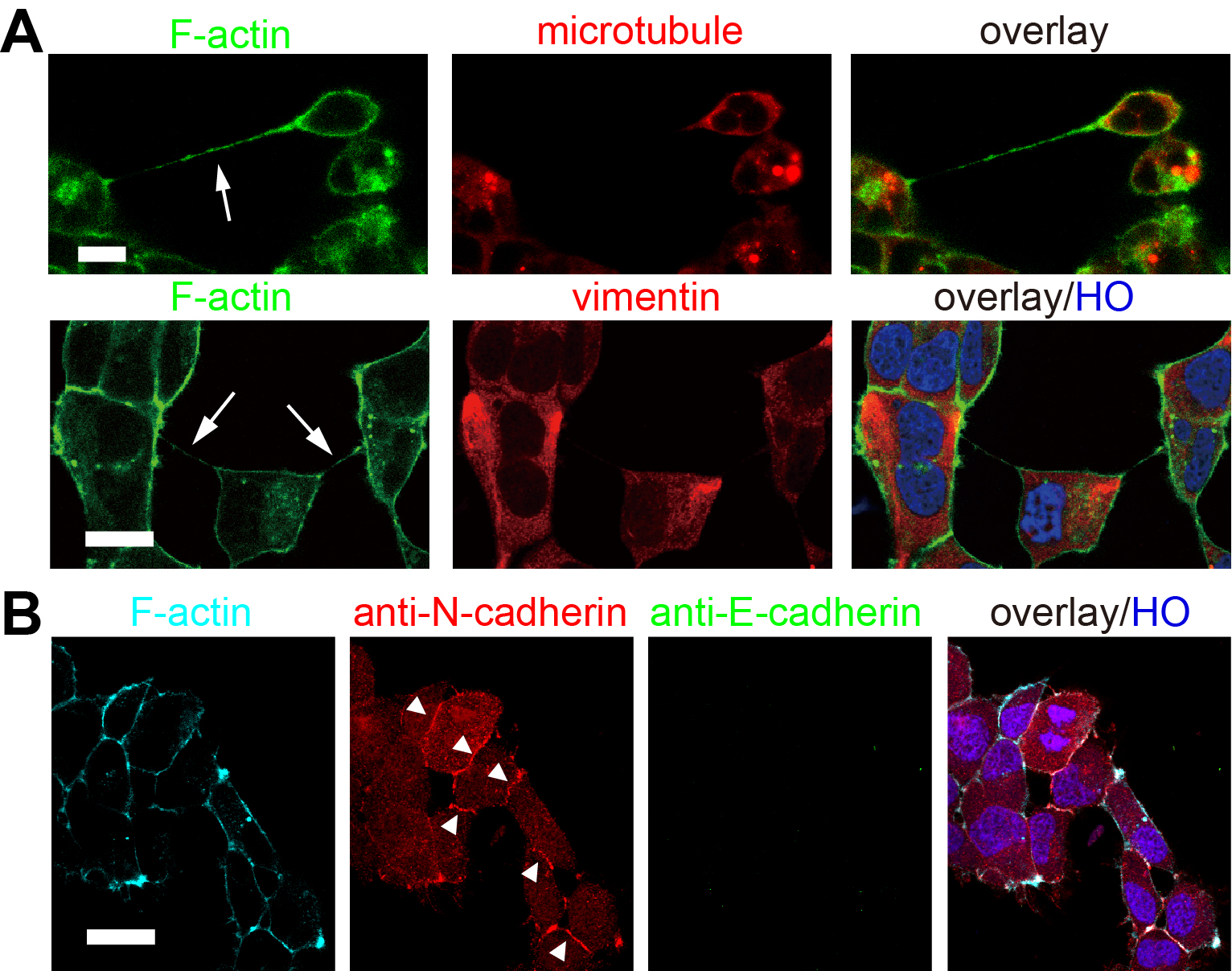

Supplement: Supplementary file 2 [file Image3.JPEG]

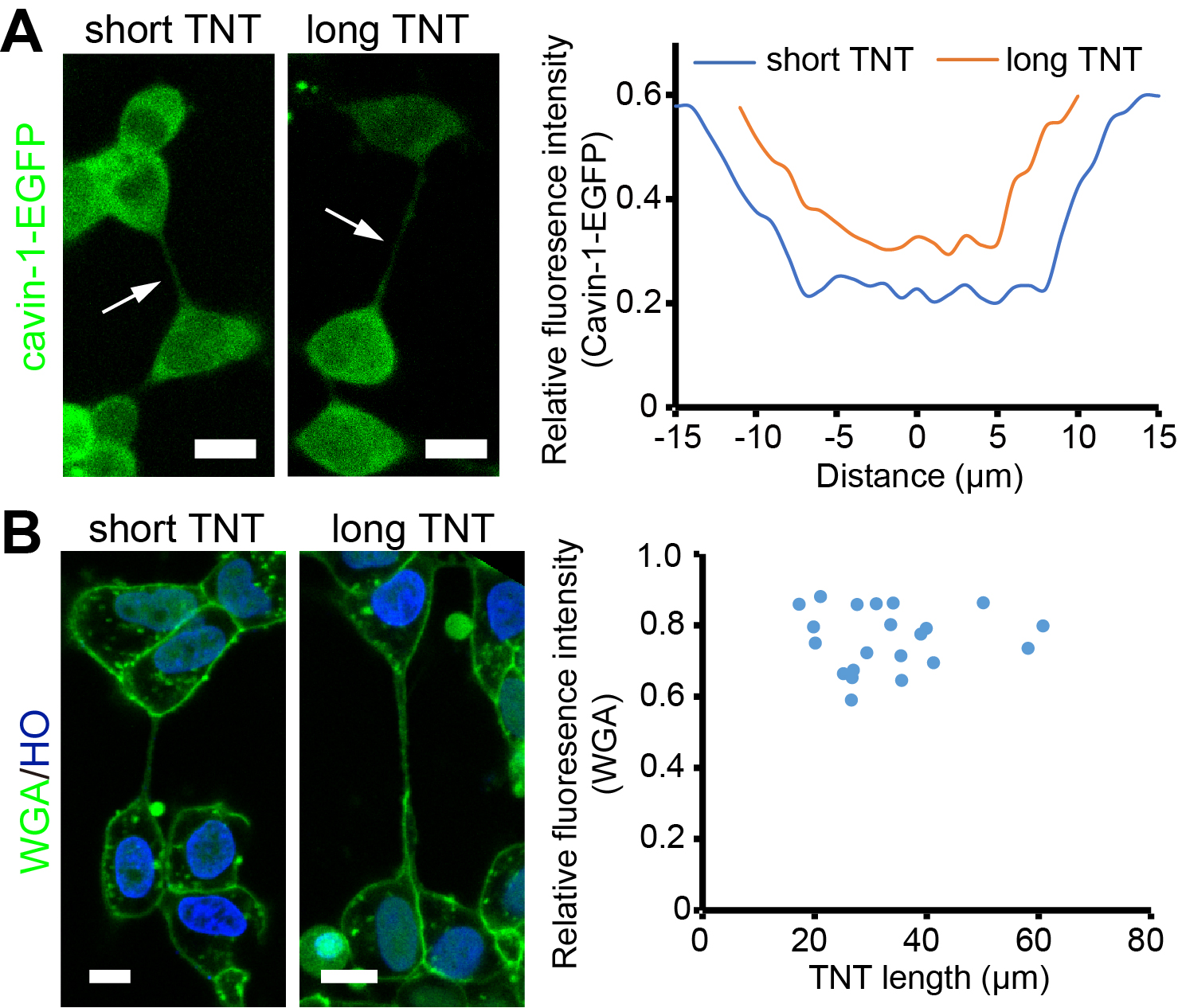

Supplement: Supplementary file 3 [file Image1.JPEG]

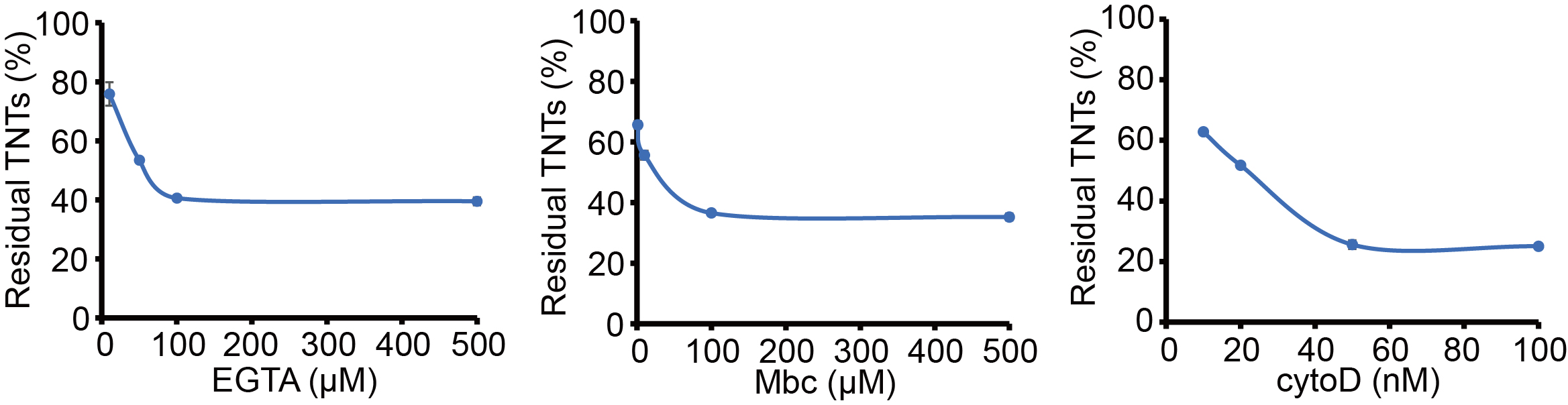

Supplement: Supplementary file 4 [file Image4.JPEG]

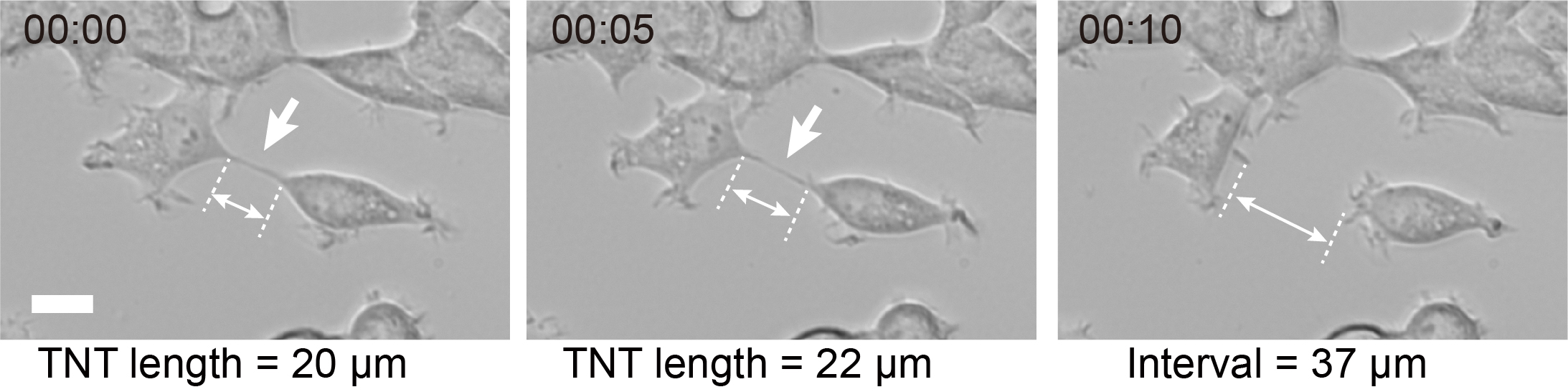

Supplement: Supplementary file 5 [file Image2.JPEG]

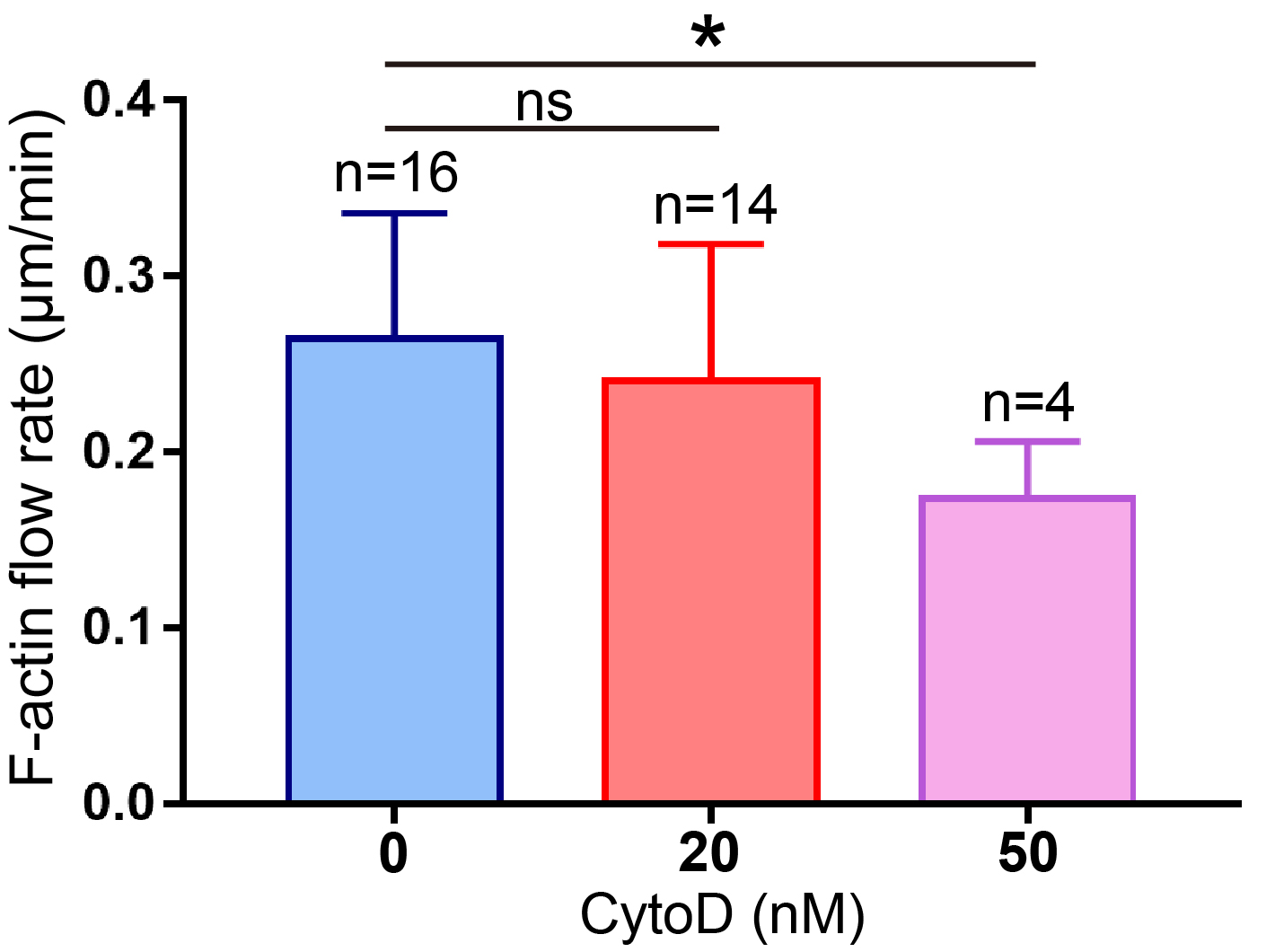

Supplement: Supplementary file 6 [file Image5.JPEG]
